# Vitiligo-specific soluble biomarkers as early indicators of response to immune checkpoint inhibitors in metastatic melanoma patients

**DOI:** 10.1038/s41598-022-09373-9

**Published:** 2022-03-31

**Authors:** Maria Luigia Carbone, Gabriele Madonna, Alessia Capone, Marianna Bove, Simona Mastroeni, Lauretta Levati, Mariaelena Capone, Paolo Antonio Ascierto, Federica De Galitiis, Stefania D’Atri, Cristina Fortes, Elisabetta Volpe, Cristina Maria Failla

**Affiliations:** 1 Experimental Immunology Laboratory, IDI-IRCCS, Via Monti di Creta 104, 00167 Rome, Italy; 2 Melanoma, Cancer Immunotherapy and Development Therapeutics Unit, Istituto Nazionale Tumori IRCCS Fondazione “G. Pascale”, Via Mariano Semmola 53, 80131 Naples, Italy; 3 Molecular Neuroimmunology Unit, IRCCS Fondazione Santa Lucia, 00143 Rome, Italy; 4 Epidemiology Unit, IDI-IRCCS, Via Monti di Creta 104, 00167 Rome, Italy; 5 Molecular Oncology Laboratory, IDI-IRCCS, Via Monti di Creta 104, 00167 Rome, Italy; 6 Oncology Department, IDI-IRCCS, Via Monti di Creta 104, 00167 Rome, Italy

**Keywords:** Biomarkers, Melanoma, Immunotherapy, Vitiligo

## Abstract

Immunotherapy with checkpoint inhibitors (CPIs) strongly improved the outcome of metastatic melanoma patients. However, not all the patients respond to treatment and identification of prognostic biomarkers able to select responding patients is currently of outmost importance. Considering that development of vitiligo-like depigmentation in melanoma patients represents both an adverse event of CPIs and a favorable prognostic factor, we analyzed soluble biomarkers of vitiligo to validate them as early indicators of response to CPIs. Fifty-seven metastatic melanoma patients receiving CPIs were enrolled and divided according to the best overall response to treatment. Patient sera were evaluated at pre-treatment and after 1 and 3 months of therapy. We found that basal CD25 serum levels were higher in stable and responding patients and remained higher during the first 3 months of CPI therapy compared to non-responders. CXCL9 was absent in non-responding patients before therapy beginning. Moreover, an increase of CXCL9 levels was observed at 1 and 3 months of therapy for all patients, although higher CXCL9 amounts were present in stable and responding compared to non-responding patients. Variations in circulating immune cell subsets was also analyzed, revealing a reduced number of regulatory T lymphocytes in responding patients. Altogether, our data indicate that a pre-existing and maintained activation of the immune system could be an indication of response to CPI treatment in melanoma patients.

## Introduction

Identification of biomarkers able to predict response to therapies and monitor disease progression is a rapidly growing field of study in cancer research. In the last decade, development of checkpoint inhibitors (CPIs) has represented the most significant breakthrough in cancer therapy, generating major clinical benefit in patients diagnosed with advanced metastatic cancer^1,2^. Despite the positive results obtained in around 40% of patients^3–5^, therapy with anti-programmed death (PD)-1 antibodies is hampered by a substantial number of unresponsive individuals. Therefore, it is now mandatory to identify prognostic biomarkers able to indicate patients who would respond to therapy, so to avoid exposure of non-responders to harmful side-effects and delay for them the introduction of alternative therapies^6–8^. Biomarkers within tumor immune microenvironment and tumor cell intrinsic features have already been studied as indicators of response to CPI therapy in metastatic melanoma. Among these, intratumor programmed death-ligand (PD-L)1 expression, number of tumor-infiltrating lymphocytes, tumor mutational burden^9–12^. However, due to intratumor heterogeneity and dynamic changes in protein expression associated with the tumor microenvironment, these tissue biomarkers obtained from the primary tumor biopsies are not appropriate for monitoring patient response during treatment. Additional biomarkers from peripheral blood samples have been studied in melanoma, e.g., neutrophil-to-lymphocyte ratio (NLR)^13,14^, serum lactate dehydrogenase (LDH) levels^15,16^, cytokines and lymphocyte populations [interleukin (IL)-6, cluster of differentiation (CD)73, CD8^+^CD73^+^ T cells]^17–19^. Data about these biomarkers could be easier obtainable and some of them have been already proposed for prognostic and diagnostic purposes, but none of them already entered the clinical practice.

During immunotherapy with CPIs, several immune-related adverse event (irAE) can be developed, especially dermatologic toxicity associated with inflammatory and autoimmune responses^20,21^. These toxicities include vitiligo-like depigmentation or leukoderma and, to a lesser extent, psoriasis, bullous pemphigoid, and lichenoid reactions^22–24^. Leukoderma development is the only irAE associated with a favorable outcome in melanoma patients receiving immunotherapy with CPIs^25–30^.

In the past, vitiligo biomarkers characterizing the vitiligo active status early on and in the absence of clinical signs of the pathology, have been identified^31^. They include an augmented presence in patient circulation of inflammatory molecules such as the chemokine (C-X-C motif) ligand (CXCL)9/MIG^32–34^, CXCL10/IP-10^32,35,36^, and CXCL11/I-TAC^36^, S100B^37,38^, IL-17A^39,40^, soluble forms of CD25/IL-2 receptor alpha (IL-2 Rα)^41–43^ and CD27/TNFRSF7^43,44^. Moreover, some microRNAs (miRNAs), such as miR-16, miR-19b, and miR-25, were highly expressed in vitiligo patients, while miR-574 was downregulated, compared with healthy controls^45–47^.

Considering that leukoderma development has been described in melanoma patients receiving CPI immunotherapy and it has been associated with a positive response to therapy^25–29,48^, we hypothesized that immunological mechanisms like those activated in vitiligo pathogenesis could be also triggered in melanoma patients responding to CPI therapy. If this would be the case, the same biomarkers that identify vitiligo active phase could also represent biomarkers of patient response to CPI therapy. In this prospective, we analyzed several biomarkers of active vitiligo in serum and plasma samples derived from metastatic melanoma patients treated with anti-PD-1 inhibitors and correlated their expression levels with patient response to therapy.

## Results

### Patients’ characteristics and responses

Fifty-seven melanoma patients were enrolled in the study. Considering the best overall response (iBOR), 28 patients responded positively to CPI therapy with an immune complete response (iCR, n = 11) or an immune partial response (iPR, n = 17), 16 patients had no objective response (immune stable disease, iSD), and 13 patients developed an immune progressive disease (iPD). The median follow-up time for progression-free survival (PFS) was 12.2 months (mean 17.1 month, ranging from 1.1 to 61.7 months). At 1 year, PFS was 57.7%, with a median PFS of 33.4 months for iCR, 20.7 for iPR, 9.7 for iSD, and 2.8 for iPD patients. There were no patients lost to follow-up. Fifteen patients developed leukoderma at a mean time of 6.8 months after starting of the CPI therapy (Table 1).

**Table 1:** Characteristics and treatment outcomes of enrolled patients.

Patient	Stage^a^	Checkpoint inhibitor^a^	iBOR^b^	PFS^c^	Progression^c^	Leukoderma^d^
1	IIIc	Nivo	iCR	10.8	No	8.7
2	M1a	Nivo	iCR	40.7	No	2.1
3	M1a	Nivo	iCR	13.5	No	–
4	M1a	Nivo	iCR	12.2	No	–
5	M1b	Pembro	iCR	21.9	No	7.6
6	M1c	Nivo	iCR	33.4	No	4.7
7	M1c	Pembro	iCR	37.3	No	11.1
8	M1c	Pembro	iCR	37.3	No	9.7
9	M1c	Pembro	iCR	48.1	No	–
10	M1c	Nivo	iCR	13.3	No	–
11	M1d	Pembro	iCR	62.6	No	–
12	M1a	Nivo	iPR	40.5	No	13.1
13	M1a	Pembro	iPR	26.2	No	–
14	M1a	Pembro	iPR	20.7	No	4.3
15	M1a	Pembro	iPR	19.1	No	–
16	M1a	Pembro	iPR	7.1	Yes	–
17	M1a	Nivo	iPR	11.5	No	–
18	M1a	Nivo	iPR	10.8	No	–
19	M1b	Pembro	iPR	33.6	No	2.1
20	M1b	Pembro	iPR	9.2	No	6.7
21	M1c	Nivo	iPR	8.9	Yes	–
22	M1c	Nivo	iPR	42.3	No	–
23	M1c	Nivo	iPR	36.8	No	9.7
24	M1c	Pembro	iPR	39.1	No	–
25	M1c	Pembro	iPR	38.0	No	7.7
26	M1c	Pembro	iPR	36.8	No	6.9
27	M1c	Nivo	iPR	13.9	Yes	–
28	M1c	Nivo	iPR	12.9	No	–
29	M1a	Pembro	iSD	7.7	Yes	–
30	M1a	Nivo	iSD	12.9	No	–
31	M1b	Nivo	iSD	18.2	Yes	–
32	M1b	Nivo	iSD	11.7	Yes	–
33	M1c	Nivo	iSD	5.9	Yes	–
34	M1c	Nivo	iSD	5.3	Yes	–
35	M1c	Nivo	iSD	36.4	No	4.3
36	M1c	Nivo	iSD	6.8	Yes	–
37	M1c	Pembro	iSD	5.2	Yes	–
38	M1c	Pembro	iSD	4.1	Yes	–
39	M1c	Pembro	iSD	32.5	Yes	–
40	M1c	Pembro	iSD	6.3	Yes	–
41	M1c	Pembro	iSD	26.4	Yes	–
42	M1c	Pembro	iSD	15.8	No	–
43	M1d	Pembro	iSD	4.8	No	2.8
44	M1d	Pembro	iSD	17.3	No	–
45	IIIc	Pembro	iPD	2.8	Yes	–
46	M1a	Pembro	iPD	2.7	Yes	–
47	M1a	Pembro	iPD	2.7	Yes	–
48	M1c	Nivo	iPD	3.6	Yes	–
49	M1c	Nivo	iPD	3.7	Yes	–
50	M1c	Nivo	iPD	4.1	Yes	–
51	M1c	Nivo	iPD	1.8	Yes	–
52	M1c	Pembro	iPD	2.0	Yes	–
53	M1c	Pembro	iPD	1.1	Yes	–
54	M1c	Pembro	iPD	4.0	Yes	–
55	M1c	Pembro	iPD	3.1	Yes	–
56	M1c	Pembro	iPD	1.4	Yes	–
57	M1c	Nivo	iPD	3.9	Yes	–

^a^Staging before starting therapy: nivo, nivolumab; pembro, pembrolizumab.

^b^iBOR, best overall response according to the iRECIST criteria: iCR, complete response; iPR, partial response; iSD, stable disease; iPD, progressive disease.

^c^Data cut off on 11th November 2020. PFS, progression-free survival (months).

^d^Patients who had developed (months) or not (–) leukoderma at the cut off data.

We then analyzed patient characteristics and tumor-associated factors at the beginning of the treatment with anti-PD-1 antibodies. As shown in Table 2, 37 patients were males (64.9%) and 20 females (35.1%). Mean age at treatment start was 67.3 years. Levels of circulating LDH were high for 13 patients (26.5%) and were not assessed for 8 patients. The M staging of the extent of metastatization was stage IIIc–M1b for 21 patients (36.8%), and M1c–M1d for 36 patients (63.2%). In particular, stage IIIc for 2 patients, stage M1a for 12 patients (M1a(0) for 8, M1a(1) for 3, LDH not assessed for 1), stage M1b for 7 patients (M1b(0) for 5, M1b(1) for 2), stage M1c for 33 patients (M1c(0) for 20, M1c(1) for 8, LDH not assessed for 5), stage M1d for 3 patients (M1d(0) for 2, LDH not assessed for 1). BRAF gene mutation was detected in 15 patients (26.8%). Thirty-one patients (54.4%) were treated with pembrolizumab and 26 patients (45.6%) with nivolumab. First-line treatment was done by 32 patients (56.1%), and second-to-fourth line by 25 patients (43.9%). Leukoderma was developed by 15 patients (26.3%). Of those, n = 6 iCR patients (40.0%), n = 7 iPR patients (46.7%), n = 2 iSD patients (13.3%), none iPD patient. Clinical characteristics of the response groups were similar for sex, age, metastasis stage, serum LDH amount, BRAF mutation, drug administrated, and line of treatment. As expected, patients who developed leukoderma were associated with a good response to CPI therapy (*p* = 0.003). Out of 15 patients with leukoderma, 12 (80%) developed it during the first-line treatment.

**Table 2:** Patient characteristics, treatments, and leukoderma development.

Characteristics	All (n = 57)	iPD (n = 13)	iSD (n = 16)	iPR (n = 17)	iCR (n = 11)	*p* value^a^
**Sex**						
Male	37 (64.9)	9 (69.2)	9 (56.3)	12 (70.6)	7 (63.6)	
Female	20 (35.1)	4 (30.8)	7 (43.7)	5 (29.4)	4 (36.4)	0.866
**Age, years**						
Mean (SD)	67.3 (12.8)	62.0 (12.0)	67.1 (13.5)	73.2 (12.2)	64.7 (11.3)	
Median (IQR)	67 (59–78)	65 (54–68)	67 (60–79)	76 (67–81)	65 (56–72)	0.084^b^
≤ 65	22 (38.6)	6 (46.1)	7 (43.7)	4 (23.5)	5 (45.4)	
> 65	35 (61.4)	7 (53.9)	9 (56.3)	13 (76.5)	6 (54.6)	0.509
**Serum LDH**						
Normal	36 (73.5)	8 (80.0)	10 (71.4)	11 (68.8)	7 (77.8)	
Elevated	13 (26.5)	2 (20.0)	4 (28.6)	5 (31.2)	2 (22.2)	0.970
**Stage**						
IIIc-M1b	21 (36.8)	3 (23.1)	4 (25.0)	9 (52.9)	5 (45.4)	
M1c-M1d	36 (63.2)	10 (76.9)	12 (75.0)	8 (47.1)	6 (54.6)	0.263
**BRAF status**						
Mutation	15 (26.8)	5 (38.5)	4 (25.0)	3 (18.7)	3 (27.3)	
No mutation	41 (73.2)	8 (61.5)	12 (75.0)	13 (81.3)	8 (72.7)	0.702
**Drug**						
Pembrolizumab	31 (54.4)	5 (38.5)	7 (43.7)	8 (47.1)	6 (54.6)	
Nivolumab	26 (45.6)	8 (61.5)	9 (56.3)	9 (52.9)	5 (45.4)	0.907
**Line of treatment**						
1	32 (56.1)	5 (38.5)	7 (43.7)	11 (64.7)	9 (81.8)	
2–4	25 (43.9)	8 (61.5)	9 (56.3)	6 (35.3)	2 (18.2)	0.117
**Leukoderma**						
No	42 (73.7)	13 (100.0)	14 (87.5)	10 (58.8)	5 (45.4)	
Yes	15 (26.3)	–	2 (12.5)	7 (41.2)	6 (54.6)	0.003

Totals may vary because missing values. Data are n (%) unless otherwise stated.

*iPD* progressive disease, *iSD* stable disease, *iPR* partial response, *iCR* complete response, *SD* standard deviation, *IQR* interquartile range, *LDH* lactate dehydrogenase.

*p* value was calculated using the Fisher's exact test (^a^) or Kruskal–Wallis test (^b^).

In a sub-group of 44 patients for whom blood analysis data were available (patients no. 1–5, 7, 10, 14–16, 18–28, 30–39, 42–44, 46, 47, 49–55, 57 of Table 1), we observed higher neutrophil count at the beginning of the therapeutic plan in iPD patients in respect with iCR patients (p = 0.043; Table 3). Moreover, our data indicated higher baseline values of NLR and derived NLR (dNLR) in iPD patients rather than in iCR patients, suggesting that these higher basal levels could associate with a tendency towards progression disease after treatment with CPIs (Table 3).

**Table 3:** Analysis of the relationship between neutrophils and lymphocytes before the start of immunotherapy with CPIs.

	iPD	iSD	iPR	iCR
Neutrophil count	5519 ± 707	5552 ± 909	4415 ± 402	3554 ± 293*
NLR	3.80 ± 0.89	3.12 ± 0.43	3.67 ± 0.50	2.25 ± 0.18
dNLR	2.44 ± 0.47	2.13 ± 0.28	2.34 ± 0.29	1.53 ± 0.09

Neutrophil count (µL); *NLR* neutrophil-to-lymphocyte ratio (neutrophils/lymphocytes), *dNLR* derived neutrophil-to-lymphocyte ratio [neutrophils/(leukocytes-neutrophils)]. Best overall response according to iRECIST criteria: iPD, progressive disease (n. 10 patients); iSD, stable disease (n. 13 patients); iPR, partial response (n. 14 patients); complete response, iCR (n. 7 patients). Data are expressed as mean value ± standard error of the mean (SEM). *p = 0.043 iCR versus iPD, assessed by Mann–Whitney U test.

### CD25 and CXCL9 as potential early biomarkers of response to CPI therapy

To search for potential biomarkers that could predict patient clinical response or that could represent early indicators of response to CPI therapy, we chose a targeted approach, selecting biomarkers of the vitiligo active phase and analyzing them in serum samples from patients before (T0) and after 1 (T1) and 3 months (T2) of treatment. We analyzed circulating levels of CD25, CD27, CXCL9, CXCL10, CXCL11, S100B, and IL-17A. In our patient subset, circulating amount of either S100B or IL-17A was below the ELISA detection level (data not shown).

**Figure 1:**
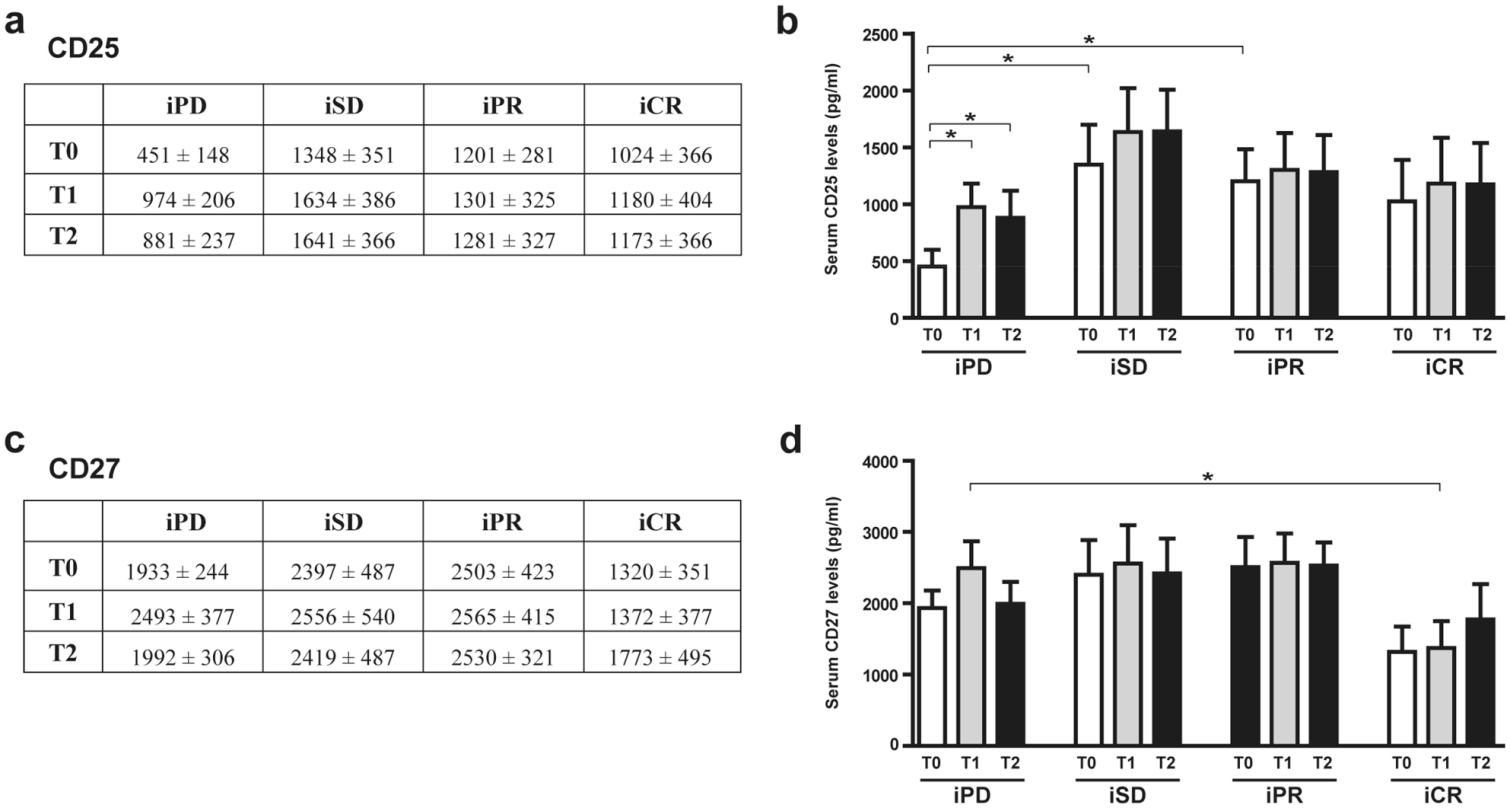
Serum levels of CD25 and CD27 in melanoma patients treated with CPIs. Protein amounts were analyzed by ELISA in serum samples of melanoma patients before (T0) and after 1 month (T1) or 3 months (T2) of treatment. Best overall response according to iRECIST criteria: iPD, progressive disease (n. 13 patients); iSD, stable disease (n. 16 patients); iPR, partial response (n. 17 patients); complete response, iCR (n. 11 patients). (**a**,**b**) CD25, (**c**,**d**) CD27 amount (pg/ml). Data are expressed as mean value ± standard error of the mean (SEM). *p < 0.05, as assessed by Mann–Whitney U test to compare between-group differences; or by Wilcoxon signed-rank test to evaluate before-after treatment differences.

Regarding CD25, iSD and iPR patients had significantly higher levels of CD25 at baseline than iPD patients (Fig. 1a,b). A tendency to higher CD25 serum amounts was observed also for iCR patients, but it did not reach statistical significance (Fig. 1a,b). Basal CD25 levels (T0) significantly increased at T1 and T2 for iPD patients, but values remained lower than for the other patient groups at the same time point.

As far as CD27, iCR patients had less circulating protein amount than other groups at all the time-point analyzed. However, statistical significance was observed only at T1 between iCR and iPD patients (Fig. 1c,d).

**Figure 2:**
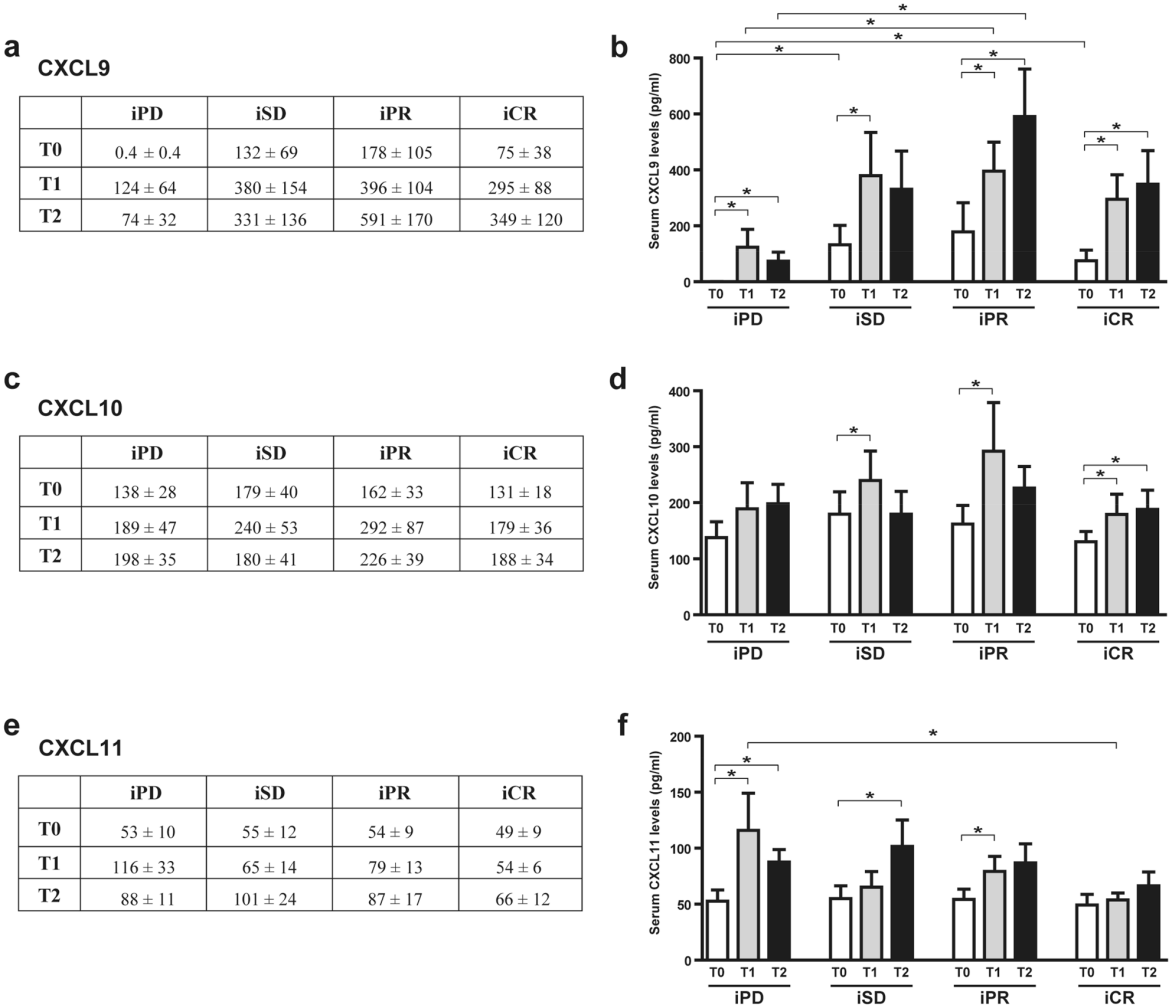
Serum levels of CXCL9, CXCL10, and CXCL11 in melanoma patients treated with CPIs. Chemokine levels were analyzed by ELISA in serum samples of melanoma patients before (T0) and after 1 month (T1) and 3 months (T2) of treatment. Best overall response according to iRECIST criteria: iPD, progressive disease (n. 13 patients); iSD, stable disease (n. 16 patients); iPR, partial response (n. 17 patients); complete response, iCR (n. 11 patients). (**a**,**b**) CXCL9, (**c**,**d**) CXCL10, (**e**,**f**) CXCL11 amount (pg/ml). Data are indicated as mean value ± standard error of the mean (SEM). *p < 0.05, as assessed by Mann–Whitney U test to compare between-group differences; or by Wilcoxon signed-rank test to evaluate before–after treatment differences.

CXCL9 was undetectable at T0 in the sera of iPD compared to other patients who had higher chemokine circulating levels at baseline (significance was achieved at T0 for iSD and iCR versus iPD patients; Fig. 2a,b). Moreover, the trend of lower circulating amount of CXCL9 in iPD patients compared to other groups was maintained after 1 and 3 months of therapy (significance was achieved at T1 and T2 for iPR versus iPD patients). The three groups of patients showed CXCL9 serum basal levels (T0) that significantly increased after 1 month of therapy (T1). Interestingly, while for iPR and iCR patients CXCL9 levels continued to significantly growth at T2, for iPD and iSD patients the levels of CXCL9 decreased at T2.

CXCL10 amounts significantly increased at T1 compared to T0 for iSD, iPR and iCR patients, and a significative increment was also present for iCR patients at T2 (Fig. 2c,d). No significant differences in serum concentration among the three response groups of patients were observed. Similarly, CXCL11 concentrations was significantly different only at T1 for iCR versus iPD patients (Fig. 2e,f). CXCL11 levels increased between T0 and T1 for iPD and iPR patients, and between T0 and T2 for iSD patients (Fig. 2e,f). In iPD patients, the increment observed at T1 was followed by a reduction in CXCL11 levels at T2 (Fig. 2e,f).

Since augmented circulating CD25 and CXCL9 levels could both represent early biomarkers of response to CPI therapy, we noticed that no correlation was present between the two proteins levels (Spearman’s ρ = 0.03, *p* = 0.821).

### Reduced amount of circulating regulatory T lymphocytes (Treg) was present in responding patients

**Figure 3:**
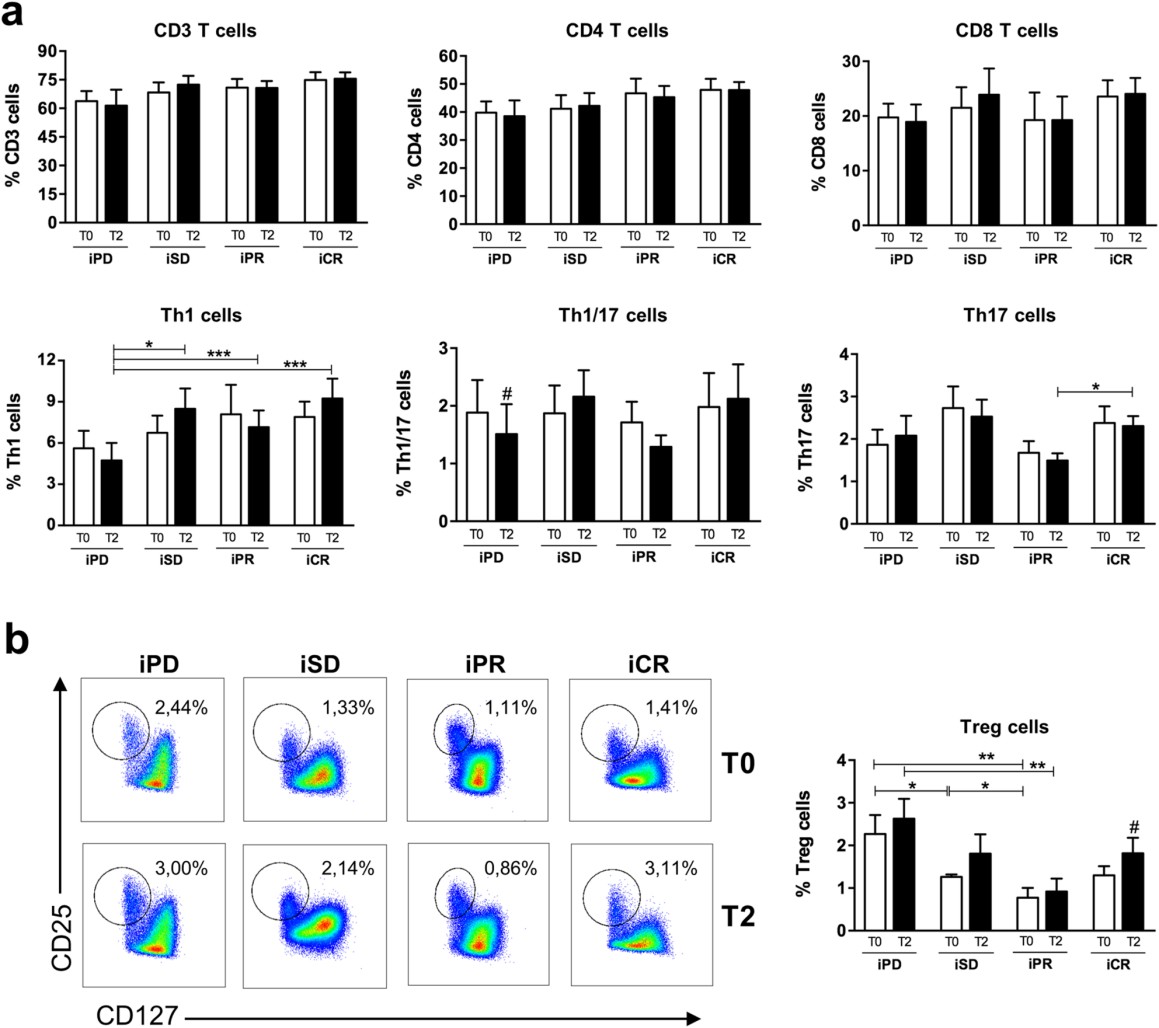
Frequency of circulating T lymphocytes in melanoma patients treated with CPIs. Frequency of circulating T lymphocytes, including (**a**) CD3, CD4, CD8, Th1, Th1/17, Th17 cells, and (**b**) Treg cells, were analyzed by flow cytometry in PBMCs of melanoma patients before (T0) and after 3 months (T2) of treatment. Representative flow cytometry plots of Treg frequency for each condition are reported in Panel (**b**). Best overall response according to iRECIST criteria: iPD, progressive disease (n. 7 patients); iSD, stable disease (n. 8 patients); iPR, partial response (n. 8 patients); complete response, iCR (n. 10 patients). Frequency data of each T cell population was calculated as percentage on total alive lymphocytes cells. Data are indicated as mean value ± standard error of the mean (SEM). *p < 0.05, **p < 0.01, ***p < 0.001 as assessed by Mann–Whitney U test to compare between-group differences; ^#^*p* < 0.05 by Wilcoxon signed-rank test to evaluate before–after treatment differences.

With the goal to understand whether the observed differences in serum levels of CD25 and CXCL9 between responding and not-responding patients could be a direct indication of immune cell activation, we measured the frequency of circulating T lymphocytes by cytofluorimetric analyses in 33 patients, for whom peripheral blood mononuclear cells (PBMCs) were available. We evaluated if T cell subtypes changed in patients before (T0) and after 3 months (T2) of CPI therapy. Patients no. 1, 3–11, 14–17, 20, 24, 27–28, 30, 32–35, 42–47, 50–51, 56–57 of Table 1 were examined (iPD, 7 patients; iSD, 8 patients; iPR, 8 patients; iCR, 10 patients). As shown in Fig. 3a and in Supplementary Fig. 1, no relevant differences in the amount of CD3^+^, CD4^+^, CD8^+^, and specific T helper cell subtypes was observed in the comparisons between patient groups at T0. T lymphocyte frequency did not change at T2, except for T helper 1 lymphocytes whose frequency is significantly lower in iPD patients than in other groups after 3 months of CPI therapy. Instead, a higher frequency of Treg was observed in iPD compared to iSD, iPR and iCR patients at T0 (Fig. 3b and Supplementary Fig. 1). This difference was maintained after 3 months of CPI therapy.

**Figure 4:**
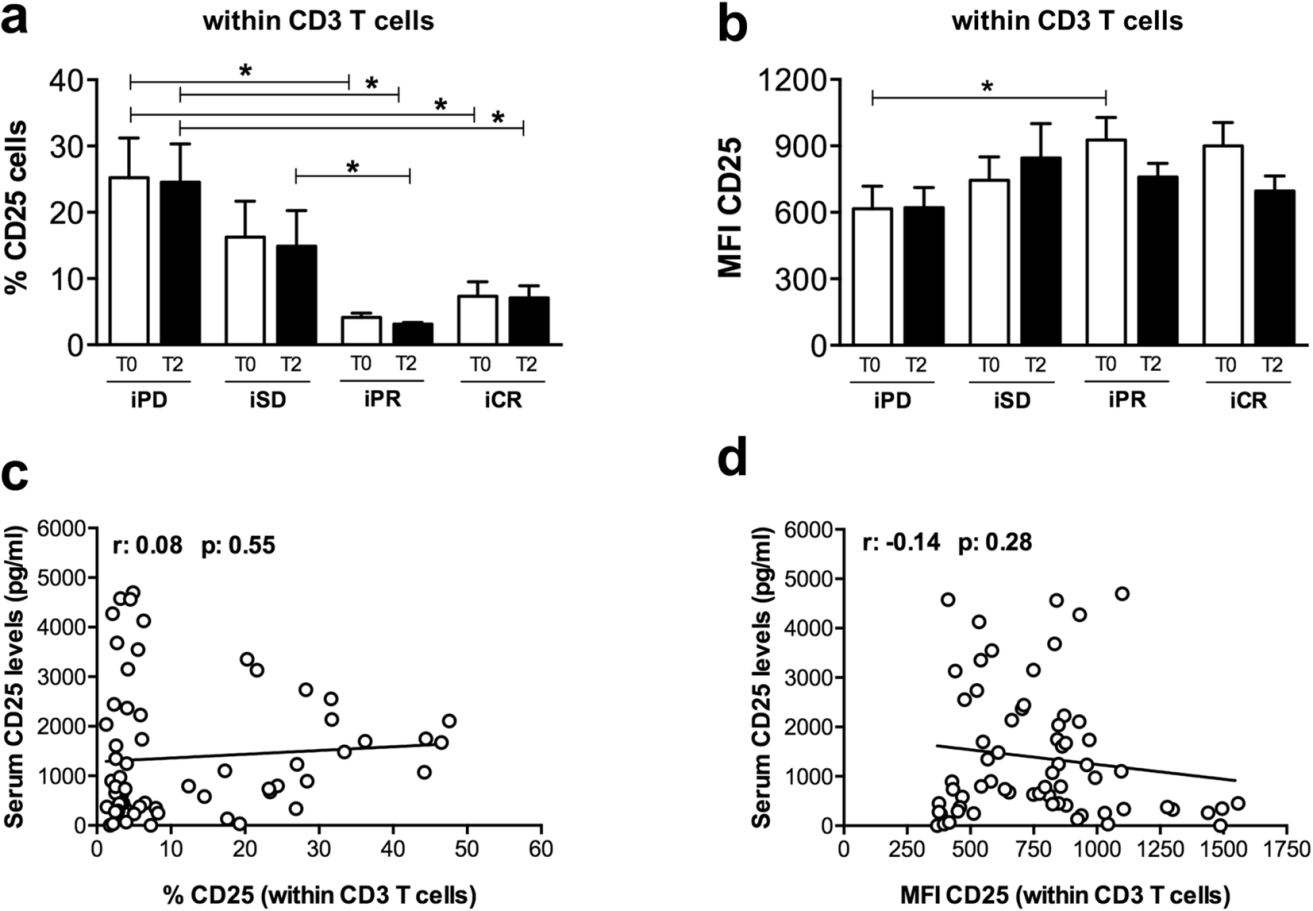
Percentage and expression of CD25 by CD3 T lymphocytes of melanoma patients treated with CPIs. Frequency (**a**) and median fluorescent intensity (MFI) (**b**) of CD25 were analyzed by flow cytometry in PBMCs within CD3 T cell population of melanoma patients before (T0) and after 3 months (T2) of treatment. Pearson’s correlation analysis between serum CD25 levels (pg/ml) and frequency of CD3^+^ CD25^+^ T cells (**c**), or MFI of CD25 within CD3 T cells (**d**) was shown. Best overall response according to iRECIST criteria: iPD, progressive disease (n. 7 patients); iSD, stable disease (n. 8 patients); iPR, partial response (n. 8 patients); complete response, iCR (n. 10 patients). (**a**,**b**) Data are indicated as mean value ± standard error of the mean (SEM). *p < 0.05 as assessed by Mann–Whitney U test to compare between-group differences.

Then, we analyzed if expression of the CD25 receptor changed on CD3^+^ T cell membrane during CPI treatment. We observed that iPR and iCR patients compared to iSD and iPD had a lower percentage of circulating CD3^+^ CD25^+^ cells at T0 and T2 (Fig. 4a), while no relevant changes were observed in CD25 fluorescence intensity (Fig. 4b). Moreover, there was no correlation between soluble CD25 serum levels and the expression of CD25 membrane receptor in CD3^+^ T cells (Fig. 4c,d).

**Figure 5:**
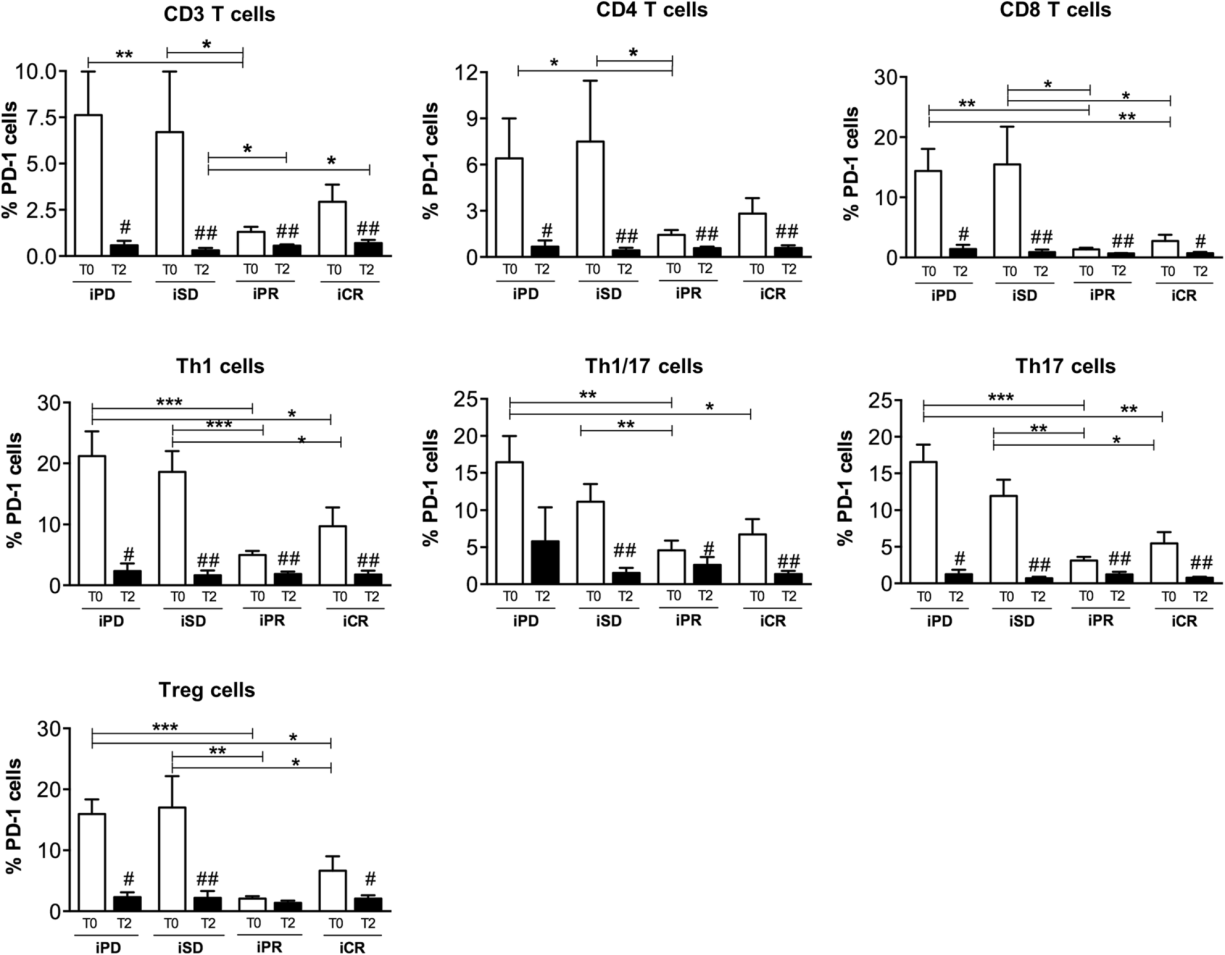
Expression of PD-1 in circulating T lymphocytes of melanoma patients treated with CPIs. Frequency of PD-1 positive cells gated within CD3, CD4, CD8, Th1, Th1/17, Th17, and Treg cells, was analyzed by flow cytometry in PBMCs of melanoma patients before (T0) and after 3 months (T2) of treatment. Best overall response according to iRECIST criteria: iPD, progressive disease (n. 7 patients); iSD, stable disease (n. 8 patients); iPR, partial response (n. 8 patients); complete response, iCR (n. 10 patients). Data are indicated as mean value ± standard error of the mean (SEM). *p < 0.05, **p < 0.01, ***p < 0.001 as assessed by Mann–Whitney U test to compare between-group differences; ^#^*p* < 0.05, ^##^*p* < 0.01 by Wilcoxon signed-rank test to evaluate before–after treatment differences.

Finally, to analyze T lymphocyte subtypes potentially responding to anti-PD-1 immunotherapy, we analyzed PD-1 expression. As shown in Fig. 5, every T cell subset examined expressed PD-1 at T0, and expression was reduced during therapy (T2). Interestingly, we observed higher percentage of PD-1^+^ cells in iPD and iSD compared to iPR and iCR patients before therapy (Fig. 5).

### miR-19b, miR-25 and miR-16 as promising indicators of response to CPIs therapy

**Figure 6:**
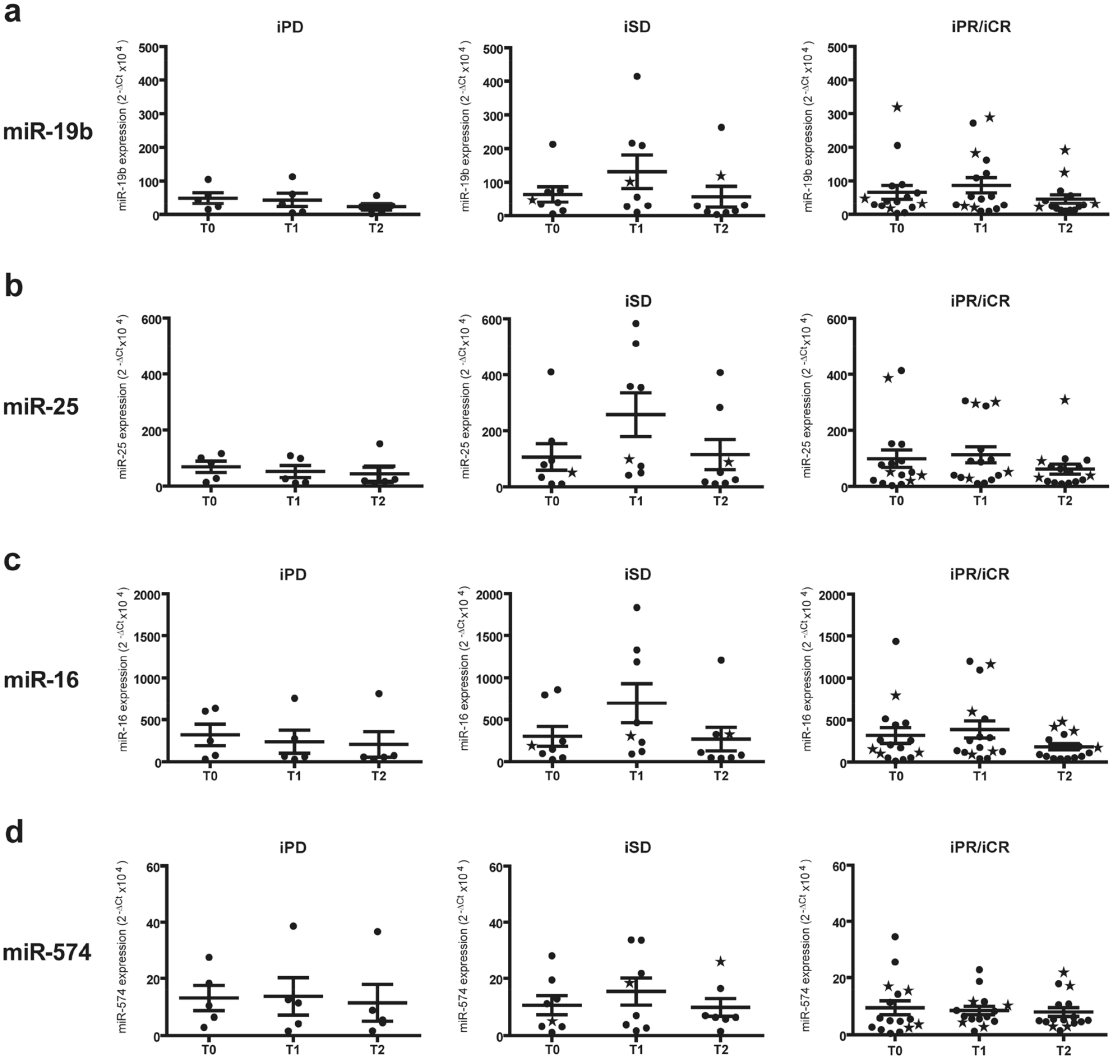
Plasma miRNA expression in melanoma patients treated with CPIs. Quantitative RT-PCR analysis of (**a**) miR-19b, (**b**) miR-25, (**c**) miR-16, and (**d**) miR-574 expression in plasma samples obtained from melanoma patients, before (T0) and after 1 month (T1) and 3 months (T2) of treatment. Best overall response according to iRECIST criteria: iPD, progressive disease (n. 5 patients); iSD, stable disease (n. 8 patients); iPR/iCR, partial response/immune complete response (n. 16 patients). The data were normalized to the level of cel-miR-39 in each sample and expressed as 2^−ΔCt^$\times$10^4^ values. Dots represent each patient values; largest bars, arithmetic mean; small bars, SEM. Stars indicate patients who had developed leukoderma at the data cut off.

For a subset of 29 patients, for whom plasma samples were available, circulating miRNAs, previously identified as biomarkers of vitiligo, were analyzed. Patients no. 1, 3–5, 9–11, 13–18, 20, 27–30, 32, 40–47, 56, 57 of Table 1 were examined (iPD, 5 patients; iSD, 8 patients; and iPR/iCR, 16 patients; iPR and iCR were assembled due to the small number of patients analyzed). As shown in Fig. 6 and in Supplementary Table 1, although statistical significance was not achieved in the comparisons between patient groups or times of drugs administration, some trends towards significance were observed. Higher levels of miR-19b and miR-25 were present at all time points of treatment in the plasma of iSD and iPR/iCR patients in respect to iPD individuals. For miR-16, a higher amount was present in iSD patients at T1 ant T2 and in iPR/iCR patients at T2 compared to iPD patients at the corresponding time points. Furthermore, miR-19b, miR-25, and miR-16 showed an increment at T1 in iSD and iPR/iCR patients, but not in iPD patients. No major differences in the amount of miR-574 among the three patient groups and at any time of treatment were observed. Interestingly, regarding miR-19b, miR-25 and miR-16, most of the iPR/iCR outliers that fall outside the distribution of miRNA levels, were the same patients who developed leukoderma during CPI therapy (stars in Fig. 6).

## Discussion

Due to the observed lack of response to CPI treatment in almost a half of melanoma patients, it is imperative to find out non-invasive indicators able to predict melanoma patient primary or secondary resistance to immunotherapy. These biomarkers of response to therapy would permit to better address melanoma patients towards the most efficacious treatments. In this regard, the intent of our work was the identification of predictive and/or early biomarkers of response to anti-PD-1 antibodies, taking advantages of previously identified biomarkers of an active vitiligo disease, since vitiligo-like leukoderma represents an irAE that often occurs during CPI therapy in melanoma patients and correlates with a positive outcome^48^. In fact, we confirmed also in our melanoma patient subset the association between development of leukoderma and a favorable clinical response.

Data we obtained examining patient sera suggest that, among the molecules that characterize the active phase of vitiligo, CD25 and CXCL9 represent independent predictive biomarkers of response to anti-PD-1 treatment. In fact, higher levels of these proteins were observed at baseline (T0) in the serum of patients who respond positively or stably to CPI therapy compared to patients who advanced towards progressive disease. In addition, a constant increment of CXCL9 circulating amounts during the therapeutic period could represent an early biomarker of response to anti-PD-1 therapy.

CD25 is the membrane IL-2 Rα that can be shed into a soluble molecule upon cell activation. CD25 plays a role in the regulation of T cell function and in the maintenance of immune tolerance^49,50^. Blockage of PD-1/PD-L1 interaction leads to an early activation of T cells, inducing several distinct signal transduction pathways involved in IL-2 expression^51^. When high levels of IL-2 are available, cells that express CD25, i.e. natural killer cells and T lymphocytes, will be activated, leading to an important immune response towards the tumor. Indeed, treatment with IL-2 was the first immunotherapy approach used in advanced melanoma. However, IL-2 benefits were associated with substantial toxicity^52–54^. In our study, we showed that stable or responding patients had higher levels of soluble CD25 at baseline and in the first months of anti-PD-1 therapy compared with patients who did not respond to treatment. Thus, we hypothesize that an increment of serum CD25 in melanoma patients who respond to anti-PD-1 therapy could be an indicator of an IL-2-mediated T cell activation, predictive of an effective anti-tumor immune response^51,55^. In future studies, it would be interesting to deeply analyze the mechanism of action of the CD25/IL-2 pathway and its time schedule in the response to anti-PD-1 treatment, and to evaluate possible beneficial effects of a combined therapy of IL-2 together with the anti-PD-1 treatment.

Chemokines play key roles in the recruitment of effector T cells into the tumor^56^. In particular, CXCR3, the receptor for the interferon-inducible chemokines CXCL9, CXCL10, and CXCL11, is highly expressed on activated T cells and plays an essential role in T lymphocyte migration into lymphoid and peripheral tissues^57–59^. A few studies have examined circulating expression of CXCR3 ligands during CPI therapies^6,8,60^. An increase of circulating CXCL9 and CXCL10 in melanoma patients was already reported during anti-PD-1 therapy^61^ and was correlated with treatment outcome^62^. In our study, we observed that, during anti-PD-1 immunotherapy, CXCL9 increased in the serum of responding and stable patients compared to non-responders, suggesting that this chemokine could be an early indicator of a positive therapeutic response. Moreover, circulating CXCL9 was absent in non-responding patients before the beginning of the treatment and increased after anti-PD-1 treatment in the sera of responding and stable patients, suggesting that a high amount of CXCL9 is important to mount an effective T cell-mediated antitumor immune response. It is worth of note that CXCL9 tissue expression was associated with CD8^+^ T lymphocyte infiltration in different solid tumors, underlining the importance of such a chemokine for an effective anti-tumor immune response^63^. It would be interesting to study if patient pretreatment with a compound able to specifically induce CXCL9 expression could be helpful in ameliorating response to anti-PD-1 antibodies as already shown in the mouse model^62^.

Our data also indicate that CXCL10 significantly increases in stable and responding patients after the first month of therapy, suggesting that an initial activation of the immune system would be fundamental for effective treatment response and tumor clearance. Conversely, elevated CXCL11 levels in blood have been previously linked to poorer outcome for CPI-treated melanoma patients^64^. CXCL11 has distinct immunoregulatory functions from those of CXCL9 and CXCL10^65^. CXCL11 limits T cell effector functions through induction and/or recruitment of Treg, and it is capable of binding to CXCR7, commonly associated with tumor cells growth and invasiveness^65,66^. Indeed, we also observed that CXCL11 significantly increased in non-responding compared to responding patients after one month of CPI therapy.

Assessment of circulating T lymphocyte subsets showed that higher frequency of Th1 cells and fewer Treg cells were indicators of a positive response to anti-PD-1 therapy. These differences were maintained even after the first months of CPI treatment. Actually, Th1 cells have recognized anticancer properties, while Treg cells are involved in tumor development and progression by inhibiting antitumor immunity^67,68^.

We also showed that PD-1 was present on all the circulating T cell subsets analyzed and that its levels significantly decreased during treatment, indicating a generalized cell response to anti-PD-1 therapy. Interestingly, we observed lower PD-1 expression in T lymphocytes of responding compared to stable and non-responding patients. Therefore, PD-1 expression could be further investigated as a potential predictive biomarker of response to CPI treatment in melanoma.

Other potential biomarkers of response to CPIs could be represented by miRNAs. Expression profiles of miRNAs are frequently dysregulated during cancer progression, and they are also involved in the modulation of the immune microenvironment^69,70^. Altered expression of miRNAs frequently correlates with poor prognosis and/or inadequate response to treatments in all stages of melanoma progression^71–73^. Several circulating miRNAs have already been described as potential prognostic biomarkers in melanoma^74–76^. In this work, we decided to analyze those miRNAs that characterize the autoimmune vitiligo disease. We showed that miR-16, miR-19b, miR-25 levels had the tendency to increase in stable and responding melanoma patients, but not in non-responder ones. Moreover, in agreement to published data^77^, we observed that circulating miR-16 levels tended to decrease in non-responding melanoma patients after CPI therapy. Lack of statistical association in our study may be due to the low number of samples analyzed that will be increased in future investigations.

Despite the presence of literature data on the role of miR-19b and miR-25 in melanoma cells^78–80^, their expression levels have not been previously analyzed in patient plasma. Therefore, for the first time, our data indicate that in metastatic melanoma patients the levels of miR-19b and miR-25 increase in stable and responding patients after one month of CPI therapy. Most responding patients with high plasma levels of miR-19b, miR-25 and miR-16 were the same patients who developed leukoderma, further implying existence of a connection between the molecular mechanisms that drive the autoimmune responses in vitiligo and the immune-mediated mechanisms that lead to an effective response to CPI therapy.

Considering patient characteristics already described as potential biomarkers of response to immunotherapy with CPIs, we also confirmed in our patient subset that basal higher neutrophil count, NLR, and dNLR could be indicators of disease progression during CPI treatment^13,14^.

In conclusion, our data confirm that leukoderma is associated with a favorable outcome in metastatic melanoma patients treated with CPIs and indicate that biomarkers of the active phase of vitiligo could represent promising indicators of response to CPIs. When validated in a larger cohort, dosage of CD25 and CXCL9 circulating levels before and in the first months of therapy could represent a tool for predicting patient response to anti-PD-1 antibodies. Both CD25 and CXCL9 should be further investigated as therapeutic targets to overcome resistance to CPI treatment in metastatic melanoma.

## Methods

### Patients, treatment, and clinical assessment

This study was conducted according to the Good Clinical Practice Guidelines and the Declaration of Helsinki. The study was approved by the Institutional Review Boards of Istituto Dermopatico dell’Immacolata (IDI)-IRCCS (510/3, April 2018), and Istituto Nazionale Tumori-IRCCS, Fondazione “G. Pascale” (20/14 oss, July 2014). All patients enrolled in the study provided written informed consent. This study included fifty-seven patients with unresectable metastatic melanoma, stage IIIc or IV based on American Joint Committee on Cancer (AJCC, 8th edition) staging^81^, enrolled for treatment with CPIs (anti-PD-1) at IDI-IRCCS or Istituto Nazionale Tumori-IRCCS, Fondazione “G. Pascale” since June 2014. Patient characteristics, blood analysis, clinicopathologic and demographic information were collected. Peripheral blood samples have been sequentially collected before therapy onset and up to one-year therapy, disease progression or leukoderma appearance. Baseline evaluation included medical history, physical examination, and radiologic tumor assessment with computed tomography scans. Nivolumab (Opdivo) was given at the dose of 240 mg every 2 weeks or 480 mg every 4 weeks, pembrolizumab (Keytruda) at the dose of 200 mg every 3 weeks. Patients underwent physical examination and assessment of biochemical parameters monthly, whereas investigator-determined objective response was assessed radiologically with computed tomography scans approximately every 12 weeks after treatment initiation. Tumor response was classified as iCR, iPR, iSD, or iPD, according to the immune response evaluation criteria in solid tumors (iRECIST)^82,83^. iPD includes both the unconfirmed progressive disease, a pseudo progression response to be confirmed in a re-evaluation follow-up visit after 4–8 weeks and confirmed progressive disease^83^. Therapy efficacy evaluation was based on iBOR and PFS. iBOR was determined as best time point response according to iRECIST. PFS was defined as time from the anti-PD-1 treatment start until diseases progression or death; if no such an event occurred, the closing date was November 11th, 2020.

### Serum preparation and molecules quantification by ELISA

Total blood samples were collected into vacutainer tubes (cat. no. 366881, BD Biosciences, Plymouth, UK), allowed to clot for 1 h at 37 °C, and centrifuged for 15 min at 1700 rcf at 4 °C. Then, serum was aliquoted and stored at − 80 °C until use. Concentrations of human CD25/IL-2 Rα (cat. no. DY223, R&D Systems, Minneapolis, USA), CD27/TNFRSF7 (cat. no. DY382-05, R&D Systems, Minneapolis, USA), IL-17A (cat. no. DY317, R&D Systems, Minneapolis, USA), S100B (cat. no. DY1820-05, R&D Systems, Minneapolis, USA), CXCL9/MIG (cat. no. DY392, R&D Systems, Minneapolis, USA) and CXCL11/I-TAC (cat. no. DY672, R&D Systems, Minneapolis, USA) were measured using DuoSet ELISA kits (R&D Systems, Minneapolis, USA). CXCL10/IP-10 levels were evaluated with a BD Pharmingen kit (cat. no. 550926, San Diego, USA). The plates were analyzed in an ELISA iMark Microplate Reader (Bio-Rad, California, USA).

### PBMCs isolation and flow cytometry analysis

Whole blood samples were collected into vacutainer sodium citrate tubes (cat. no. 367704, BD Biosciences, Plymouth, UK) and PBMCs were isolated by Ficoll gradient centrifugation (GE Healthcare, Little Chalfont, UK). Cryopreserved PBMCs were stained with the following antibodies: Panel 1: anti-human CD4 FITC (1:100) (cat. no. 130-114-531, Miltenyi Biotec, Auburn, CA, USA) (1:100), anti-human CRTh2-PE (1:150) (cat. no. 130-114-128, Miltenyi Biotec, Auburn, CA, USA), anti-human CD161-PE/Dazzle594 (1:50) (cat. no. 339939, Biolegend, San Diego, CA, USA), anti-human CD3 PercP-Cy5.5 (1:300) (cat. no. 300327, Beckman Coulter, Brea, CA, USA), anti-human CXCR3-APC Alexa647 (1:40) (cat. no. 353711, Biolegend, San Diego, CA, USA), anti-human CD8-APC Alexa700 (1:120) (cat. no. A66332, Beckman Coulter, Brea, CA, USA), anti-human CCR6-BV421 (1:30) (cat. no. 353407, Biolegend, San Diego, CA, USA), anti-human PD1-BV650 (1:30) (cat. no. 564104, BD Biosciences, Plymouth, UK), and LIVE/DEAD™ Fixable Aqua Dead Cell Stain Kit (1:200) (cat. no. l34957, Invitrogen, Waltham, MA, USA). Panel 2: anti-human CD4 FITC (1:100) (cat. no. 130-114-531, Miltenyi Biotec, Auburn, CA, USA), anti-human CD3-ECD (1:100) (cat. no. IM2705U, Beckman Coulter, Brea, CA, USA), anti-human CD127-APC Alexa700 (1:200) (cat. no. 351343, Beckman Coulter, Brea, CA, USA), anti-human CD25-BV421 (1:60) (cat. no. 564033, BD Biosciences, Plymouth, UK), anti-human PD1-BV650 (1:30) (cat. no. 564104, BD Biosciences, Plymouth, UK), and LIVE/DEAD™ Fixable Aqua Dead Cell Stain Kit (1:200) (cat. no. l34957, Invitrogen, Waltham, MA, USA). Samples were acquired using Cytoflex cytometer (Beckman Coulter, Brea, CA, USA) and analyzed using FlowJo-10 software version 10.3.0.

### Circulating total RNA extraction and quantitative miRNA real-time RT‑PCR

Total blood samples were collected into vacutainer sodium citrate tubes, centrifuged twice for 10 min at 1260 rcf at 4 °C, and the plasma was aliquoted and stored at − 80 °C. Total RNA was extracted and purified from 200 μl of plasma using a miRNeasy Serum/Plasma kit (QIAGEN, Hilden, Germany), according to manufacturer’s instructions. A fixed volume of 2 μl of total RNA was used as input for consecutive reactions including poly(A) tailing, ligation, reverse transcription, and miR-Amp reaction with a TaqMan Advanced miRNA cDNA Synthesis kit (Applied Biosystems, CA, USA). The miRNA levels were then assessed by TaqMan Advanced miRNA Assay with TaqMan Fast Advanced miRNA master mix (Applied Biosystems, CA, USA). Each PCR plate was run in the QuantStudio 5 Real-Time PCR System (Applied Biosystems, CA, USA) according to manufacturer’s recommendations. The TaqMan Advanced miRNA probes (5′-phosphorylated) were as follows: hsa-miR-19b-3p (ID 478264_mir), hsa-miR-25-3p (ID 477994_mir), hsa-miR-16-5p (ID 477860_mir), hsa-miR-574-3p (ID 478163_mir). For quality control and analyses of miRNA expression, cel-miR-39-3p (ID 478293_mir, cat. no. A25576, Applied Biosystems, CA, USA) was used as an exogenous spike-in. Expression of miRNAs relative to cel-miR-39-3p was determined using the formula 2^−ΔCt^, where ΔC_T_ = C_TmiRNA_ − C_TmiR-39_, and C_T_ (i.e., threshold cycle) indicates the fractional cycle number at which the amount of amplified target reaches a fixed threshold. To simplify data presentation, the relative expression values were multiplied by 10^4^. All determinations were performed at least three times, each in duplicate.

### Statistical analysis

Groups based on treatment-response were compared using the Fisher's exact test for categorical variables, the non-parametric Mann–Whitney U test or the Kruskal–Wallis test for continuous variables. The Spearman’s coefficient was used to test correlation between levels of analyzed molecules at baseline. To evaluate before–after treatment differences, the Wilcoxon matched-pairs signed-rank test was used. For data analysis, the value of 0.01 pg/ml was assigned to samples with values under the lower limit of ELISA detection.

Statistical significance was set at *p* < 0.05. All statistical analyses were conducted using GraphPad prism Software (La Jolla, CA, USA).

## Supplementary Information

Supplementary Information.

## Data Availability

All data generated or analyzed during this study are available from the corresponding author on reasonable request.

## Abbreviations

CPIs: Checkpoint inhibitors
PD-1: Programmed death-1
PD-L1: Programmed death-ligand 1
NLR: Neutrophil-to-lymphocyte ratio
LDH: Serum lactate dehydrogenase
irAE: Immune-related adverse event
CXCL: (C-X-C motif) ligand
IL: Interleukin
CD: Cluster of differentiation
IL-2 Rα: IL-2 receptor alpha
miR: MicroRNA
AJCC: American Joint Committee on Cancer
iCR: Complete response
iPR: Partial response
iSD: Stable disease
iPD: Progressive disease
iRECIST: Immune response evaluation criteria in solid tumors
iBOR: Best overall response
PFS: Progression-free survival
dNLR: Derived NLR
Th: T helper lymphocytes
Treg: Regulatory T lymphocytes
PBMCs: Peripheral blood mononuclear cells
