# Supplementary material for: Vitiligo-specific soluble biomarkers as early indicators of response to immune checkpoint inhibitors in metastatic melanoma patients
Source: Sci Rep. 2022 Mar 31;12:5448. doi: 10.1038/s41598-022-09373-9 (PMC8971439; doi:10.1038/s41598-022-09373-9)
Supplement: Supplementary file 1 — Supplementary Information. [file 41598_2022_9373_MOESM1_ESM.doc]

**Supplementary figure 1.** Cryopreserved PBMCs were stained with specific antibodies and analyzed by flow cytometry. Plots represent the gating strategy used to evaluate the frequency of circulating T lymphocytes, including CD3, CD4, CD8, Th1, Th1/17, Th17 (a), and Treg (b) populations.

**
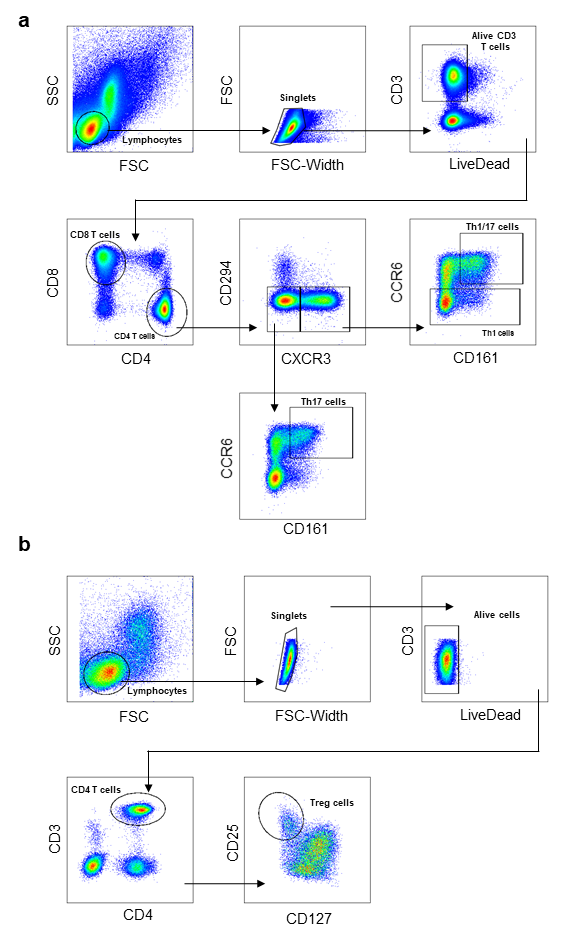
**

**Supplementary table 1. Expression of miRNA in the plasma of melanoma patients treated with CPIs.**

|  | | **iPD** | | | **iSD** | | | **iPR/iCR** | | |  | |
| --- | --- | --- | --- | --- | --- | --- | --- | --- | --- | --- | --- | --- |
|  | |  | **^a^*p*** | **^b^*p*** |  | **^a^*p*** | **^b^*p*** |  | **^a^*p*** | **^b^*p*** | **^c^*p*** | **^d^*p*** |
| **miR-19b** | T0 | 49 ± 16 | *0.813* | *0.188* | 64 ± 23 | *0.250* | *0.547* | 66 ± 20 | *0.816* | *0.421* | *0.943* | *0.967* |
|  | T1 | 44 ± 20 |  |  | 131 ± 50 |  |  | 87 ± 23 |  |  | *0.284* | *0.433* |
|  | T2 | 23 ± 10 |  |  | 57 ± 31 |  |  | 46 ± 13 |  |  | *0.724* | *0.257* |
| **miR-25** | T0 | 69 ± 20 | *0.813* | *0.438* | 107 ± 47 | *0.109* | *0.844* | 99 ± 31 | *0.623* | *0.093* | *0.943* | *0.837* |
|  | T1 | 52 ± 21 |  |  | 258 ± 78 |  |  | 113 ± 29 |  |  | *0.065* | *0.386* |
|  | T2 | 44 ± 27 |  |  | 115 ± 54 |  |  | 62 ± 18 |  |  | *0.435* | *0.386* |
| **miR-16** | T0 | 318 ± 128 | *1.000* | *0.813* | 300 ± 117 | *0.109* | *0.844* | 316 ± 94 | *0.660* | *0.266* | *0.943* | *0.837* |
|  | T1 | 238 ± 137 |  |  | 696 ± 234 |  |  | 387 ± 101 |  |  | *0.093* | *0.342* |
|  | T2 | 206 ± 152 |  |  | 267 ± 140 |  |  | 180 ± 37 |  |  | *0.622* | *0.433* |
| **miR-574** | T0 | 13 ± 4 | *0.813* | *0.625* | 11 ± 3 | *0.250* | *0.938* | 9 ± 2 | *0.489* | *0.623* | *0.833* | *0.265* |
|  | T1 | 14 ± 7 |  |  | 15 ± 5 |  |  | 8 ± 1 |  |  | *0.943* | *0.663* |
|  | T2 | 11 ± 6 |  |  | 10 ± 3 |  |  | 8 ± 2 |  |  | *0.755* | *0.901* |

miRNAs differentially expressed between plasma samples of melanoma patients before therapy (T0) and after 1 month (T1) or 3 months (T2) of treatment by qRT-PCR analysis. Best overall response according to iRECIST criteria: iPD, progressive disease (n. 5 patients); iSD, stable disease (n. 8 patients); iPR/iCR, partial response/immune complete response (n. 16 patients). The data were normalized to the level of *c. elegans* miR-39 in each sample and expressed as mean value (2-ΔCtx10^4^) ± standard error of the mean (SEM).

Statistical analysis assessed by Wilcoxon signed-rank test to evaluate before-after treatment differences (^a^*p* value T1 versus T0, ^b^*p* value T2 versus T0); or Mann-Whitney U test to compare between-group differences (^c^*p* value iSD versus iPD, ^d^*p* value iPR/iCR versus iPD).
